# Attitudes and psychotropic preferences of primary care providers in the management of mental disorders: a web-based survey

**DOI:** 10.3389/fmed.2024.1427745

**Published:** 2024-08-01

**Authors:** Dilek Örüm

**Affiliations:** Psychiatry, Elazığ Fethi Sekin City Hospital, Elazığ, Türkiye

**Keywords:** family medicine, primary care, prescription practice, psychotropic preferences, treatment attitude, general practitioner

## Abstract

**Background:**

Many variables may affect the approaches of primary care providers (PCPs) to mental disorders. This study was aimed at reaching PCPs actively practicing in Turkey through a web-based survey and determining their practices and attitudes regarding mental disorders.

**Methods:**

This was a web-based, quantitative, cross-sectional, primary care approach-based observational survey.

**Results:**

Data from 454 PCPs (213 male, 241 female; 321 general practitioners, 133 family medicine specialists) were examined. In-service training in psychiatry (*p* < 0.001), using classification criteria when evaluating mental disorders (*p* < 0.001), and experience in diagnosing mental disorders (*p* = 0.003) were more prevalent among family medicine specialists than general practitioners. Regardless of specialization status, PCPs reported the most difficulty diagnosing bipolar disorder (62.33%) and following-up alcohol/drug use disorder (52.20%). Significant differences in the use of psychotropic medications were observed between general practitioners and family medicine specialists. While the rate of direct referral to psychiatry without intervening in certain situations was higher among general practitioners, variety of psychotropic medication use were also more evident among them. Misinformation that antidepressants cause forgetfulness, numbness, suicide, and addiction was prevalent among all PCPs. Those who had in-service training in psychiatry had significantly more experience in using classification criteria, diagnosing and starting treatment for mental disorders, using psychotropic medications, and encountering suicide-related situations (*p* < 0.05). Binary logistic regression analysis demonstrated that psychiatry in-service training experience can improve the use of classification criteria, suicide detection, antidepressant choice in anxiety, and understanding the addictive nature of antidepressants (Sensitivity = 88.6%; Specificity = 98.3%; Beginning block −2 Log likelihood 628.946, overall *p* value < 0.001; Block one −2 Log likelihood 141.054^a^, Cox & Snell *R*^2^ = 0.659, Nagelkerke *R*^2^ = 0.878; Hosmer and Lemeshow Test *p* = 0.938).

**Conclusion:**

This study makes significant contributions to the literature by discussing the subject in detail and comparing general practitioners and family medicine specialists. Regardless of their specialty status, PCPs’ knowledge about mental disorders needs to be improved. In-service psychiatry training is one of the tools that can be used for this purpose.

## Introduction

Mental healthcare is a fundamental human right, and mental health is an integral part of life. It surrounds our entire lives by affecting our emotions, thoughts, and behaviors, and supports our social and economic development. A person with adequate mental health establishes positive relationships, achieves a sense of belonging, copes with stressful conditions, quickly adapts to new ideas and changes, receives training on various subjects, acquires new skills, becomes aware of his/her abilities, has goals, and cares about the well-being of himself/herself and others (1, 2). Conditions resulting from deterioration of mental health constitute a significant portion of the global burden of disease. According to the World Mental Health Report (3), in 2019, worldwide, one in eight people suffers from a mental disorder. Of the 970 million people living with mental disorders, 31% has anxiety spectrum disorders, 28.9% has depression spectrum disorders, 4.1% has bipolar spectrum disorders, and 2.5% has schizophrenia spectrum disorders. Suicide accounts for more than one in every 100 deaths globally and for every death by suicide there are more than 20 suicide attempts. Mental disorders are the leading cause of years lived with disability, accounting for one in every 6 years lived with disability globally. According to 2019 data, 39.0% of the global burden of mental disorders in disability-adjusted life years is depressive disorders, 22.0% is anxiety disorders, 11.7% is schizophrenia, and 6.5% is bipolar disorder (3). Overall, mental health issues have significant financial repercussions. Health care expenses are frequently far outweighed by productivity losses and other indirect costs to society (4). In this respect, the management of mental disorders has a significant effect on quality-adjusted life years (5). Although there is a bidirectional relationship between mental and physical health, prioritizing other medical diseases before mental health is a common practice, and community-based mental health care is routinely neglected in mental health budgets (6).

Diagnosis and treatment of anxiety and depression spectrum disorders, which constitute more than three-fifths of the global burden of mental disorders, are essential and important (3). Mental disorders that may present with psychotic symptoms, such as bipolar and schizophrenia spectrum disorders primarily affect the working-age population and in cases of acute exacerbation, they may cause serious harm to the individual and his/her environment (7). Suicide, affects people and their families from all countries and contexts, and at all ages, continues to be an important problem. All these psychiatric conditions are waiting to be solved for all humanity, especially middle-income countries (3).

Primary care services are the first units where people experiencing symptoms of mental disorders and patients diagnosed with mental disorders can be followed-up and treated. In addition, subthreshold symptoms of mental disorders can be detected in some patients who visit primary care providers (PCPs) with general medical conditions other than symptoms of mental disorders. Since patients with subthreshold mental disorders have poor disease perception, it is important to inquire about or screen for symptoms of mental disorders in these patients (8). Kessler et al. (9) reported that the diagnosis of mental disorders by PCPs was under 10.0%, demonstrating the need for careful evaluation of the mental status of primary care patients. In primary care, suprathreshold and subthreshold mental disorders constitute a significant portion of morbidity (8, 9). However, due to a number of circumstances, such as ignoring symptoms of mental disorders, somatic presentation of symptoms, lack of adequate training, poor levels of diagnosis by PCPs, limited access to psychosocial and pharmaceutical therapy, stigmatization, self-stigmatization, and patient overload, a sizeable portion of cases remains undiagnosed, untreated, or treated improperly (10). The increasing recognition of the impact of mental disorders on the global burden of disease has led to the development of primary care programs for mental disorder management in high-income countries. Despite model interventions, gaps in this field, which includes detection, treatment and follow-up processes, persist all over the world, even in high-income countries (11, 12). In addition to access barriers, more than 75.0% of people in low- and middle-income countries receive no treatment of mental disorders (12). Factors such as psychotropic medication selection, possible combinations, possible side effects, dosage, route and time of administration, duration of use, non-mental health diseases and interactions with medications, absence of effective collaboration between different healthcare specialists, and lack of evidence-based guideline-consistent care are the main source of gaps in the management of mental disorders (13, 14).

There are various studies examining PCP approaches to mental issues. In the majority of these studies, psychotropic-prescribing patterns of PCPs were examined using a small number of variables (15, 16). There has not been enough studies evaluating the approach of PCPs to mental disorders and psychotropic preferences together. This study, which also included the impact of in-service training in psychiatry and primary care specialization, aimed to examine the sociodemographic and educational characteristics, experience and attitudes regarding mental disorders, and psychotropic-use characteristics of PCPs actively working in Turkey through a web-based survey. Our hypothesis was that training characteristics and current working conditions of PCPs affect their approaches to mental disorder management. The results of this study may pave the way for organizing in-service training related to mental disorders for PCPs.

## Materials and methods

This was a web-based, quantitative, cross-sectional, and observational survey.

It is almost impossible to reach PCPs face to face, working anywhere in Turkey to gather their opinions on the current issue and create a road map for mental disorder management. In addition, people may avoid participating in scientific research during the day or during working time, or even if they participate, they may be careless in complying with research instructions. Internet offers various tools that we can use to overcome these difficulties that may be encountered in scientific data collection processes. Web-based survey tools such as Google Forms make it much easier to reach target groups in research. These forms can be created and applied free of charge (17). The current survey was conducted by distributing a web questionnaire to PCPs included in WhatsApp and Yahoo groups representative of the PCPs working actively in Turkey.

### Sampling frame and general information

In Turkey, one graduates as a general practitioner after 6 years of medical school education. To become a family medicine specialist, it is necessary to pass the medical specialization exam. Physicians who choose family medicine in the medical specialization exam participate in a three-year residency training period. During these 3 years, family medicine residents learn the principles of their own discipline and also rotate in the departments of internal medicine, gynecology and obstetrics, child health and diseases, general surgery, and psychiatry. At the end of this period, the family medicine resident who completed the medical specialization thesis receives the title of family medicine specialist. The population of this study included all PCPs working actively in Turkey. All PCPs included in this study were medical doctors, and clinicians actively working in family health center which is a health institution where primary care services are provided by one or more PCPs and family health personnel.

In this study, the concept of PCP refers to all general practitioners and family medicine specialists. In-service training in psychiatry does not refer to academic and paid training, but rather training provided free of charge to physicians by the state. As it is known, the names of the same medical diseases can be written differently in classification systems. Mental disorders were evaluated using the concept of spectrum in order to make it easier for PCPs who do not use a classification system or use a different classification system to understand the same thing.

In this study, experiences of PCPs in diagnosing mental disorders were inquired. The purpose here is to inquire whether the PCP has considered the diagnosis of any mental disorder. It is appreciated that it is not possible to determine the accuracy of diagnostic thought and experience retrospectively. That is, the expression “diagnosis experience” in this study was used to describe patients who were currently diagnosed with a mental disorder and patients who were considered to be diagnosed with a mental disorder by their PCP, even though they did not have a history of mental disorder.

In Turkey, PCPs provide services almost all over the country, including rural settlements such as villages and towns, while physicians who specialize in a particular field provide services in larger and urban settlements. In order to facilitate access to medical treatments for people living in rural areas, PCPs have been given a wide range of prescribing opportunities. Antidepressant (e.g., tricyclic antidepressants, selective serotonin reuptake inhibitors, serotonin-norepinephrine reuptake inhibitors, noradrenaline and specific serotoninergic antidepressants), antipsychotic (e.g., typical antipsychotics, atypical antipsychotics), benzodiazepine, and psychostimulant medications can be prescribed by PCPs. Psychostimulants such as methylphenidate are included in the red prescription drug class. Drugs with addictive effects such as biperiden and benzodiazepine are in the green prescription drug class. Long-acting injectable antipsychotic medications (e.g., haloperidol decanoate, paliperidon palmitate once monthly and three monthly, aripiprazole maintena) can be prescribed by PCPs during the maintenance treatment if reported by a psychiatry specialist.

The “*e-nabiz*” application, created by the Ministry of Health of the Republic of Turkey and made available to all physicians in Turkey, guides PCPs in follow-up and treatment processes. *e-nabiz*, the national patient registration system, is a database where all medical histories of patients (surgery, hospitalization, laboratory, imaging, allergies, diagnoses, medications, vaccination schedule, cancer screening data, intensive care information, reports, emergency notes) can be accessed (18).

In-service training is training provided to individuals who are employed and continue to work, to enable them to acquire the necessary knowledge, skills and attitudes related to their duties (19). In-service training increases the knowledge and skills of PCPs with regard to the management of mental disorders (20).

### Sample size calculation

According to the data of the Ministry of Health of the Republic of Turkey, the number of PCPs actively working in Turkey is estimated to be approximately 29,970 as of 2024 (21). This means 380 or more measurements/surveys are needed to have a confidence level of 95% that the real value is within ±5% of the measured/surveyed value (22).

### Development of questionnaire

The survey draft was created by the conductor of the study, who has 6 years of experience in psychiatry practice. All questions had neutral content and leading questions were avoided. The survey language was Turkish. While creating the survey, literature, primary care training in the Turkey, and clinical experiences were taken into consideration. The survey was piloted and further revised based on feedback from 10 PCPs. The survey, created via Google Form (Alphabet, Googleplex, Mountain View, California, United States) (17), was delivered to participants working in the Turkey via WhatsApp and Yahoo groups. WhatsApp and Yahoo groups, which are thought to have similar ones in every country, were unofficial, but they were the groups in which the majority of PCPs in Turkey participated. These groups were created by administrators of the country’s federation of primary care associations, and joining the group required a reference from an administrator in the group.

### Recruitment procedure and consent process

An initial e-mail/message and up to five e-mail/message reminders were sent to WhatsApp and Yahoo groups. In this e-mail/message, it was clearly stated that the survey aimed to reach PCPs actively working in Turkey and examine their approaches to mental disorders. In order to facilitate the participation of the sample group in the survey, it was emphasized that the estimated completion time of the survey was 4 or 5 min. Participants were directed to the Google Form research page by clicking on the “https://” link of this web-based form. The survey’s landing page included information such as the name of the survey, purpose of the study, ethics committee approval information, information that the data will be kept confidential, information that the currently working PCPs will participate in the study, the survey does not contain questions that may cause personal sensitivity, and average filling time of the survey. Following this information, the question “Do you approve of participating in this study?” was asked, and those who answered “yes” included in the study. Participants were not able to skip items except for the gender question. The survey was open from November 1, 2023 – March 31, 2024.

### Inclusion and exclusion criteria

Those who were not actively practicing primary care were not included in the study. There was no age or gender limit. Each response was evaluated on its own. It was planned to exclude discordant respondents from the study. However, it was observed that the participants answered all questions completely and harmoniously, including gender. Therefore, no data were excluded from the study.

### Dependent and independent variables

In this study, the relationship of many dependent variables including using classification criteria of psychiatry, experiences with mental disorder diagnoses, approach to the psychotic disorder, experiences of using psychotropic medications, psychotropic preferences in symptoms of mental disorders, information about antidepressants, and, independent variables including gender and specialization status were examined and the findings were demonstrated through tables.

### Ethical approval and funding

Ethical approval was obtained from the Firat University Non-invasive Research Ethics Committee and the 1964 Declaration of Helsinki was complied with (Date: 27/09/2023; Number: 2023/13-23). All respondents provided their consent for the information provided to be used for research purposes. No funding was declared.

### Statistical analysis

The web-based survey was hosted on the Google Forms platform, a secure end-to-end encrypted form builder for free to create online forms that capture classified data (17). Data was downloaded and stored on Microsoft Excel, an application for managing online surveys and databases. All analyses were performed using IBM SPSS Statistics version 22.0. Descriptive statistics and continuous variables were given as mean ± standard deviation, and categorical variables were given as frequency and percentage. The Chi-square test was used to compare the categorical data between the groups and genders. Binary logistic regression analysis was used in variable prediction. In regression analysis, the grouping variable (general practitioner & family medicine specialist or have psychiatry in-service training and do not have psychiatry in-service training) was accepted as the dependent variable, and, clinical variables as the independent variable. Binary logistic regression analysis was applied separately for each independent variable, and those with significant *p* values were included in the model. Variables that did not contribute sufficiently to the model were subsequently excluded. The suitability of the independent variable to the model was checked through the Hosmer and Lemeshov test. A *p* value of less than 0.05 was set as statistical significance.

## Results

### Sociodemographic and training characteristics of PCPs

Data from 454 PCPs (213 males, 241 females) were examined. While the mean age was (*n* = 454) 37.24 ± 3.36 years (min 26 years, max 57 years), the mean duration of primary care practice (*n* = 454) was 7.34 ± 5.58 years (min 1 year, max 25 years). There were 96 PCPs (21.14%) whose average number of admission to outpatient clinic per day was between 0 and 20, of 166 PCPs (36.56%) was between 21 and 50, and of 192 PCPs (42.30%) was more than 50. Average number of patients examined daily in outpatient clinic was similar between genders (*p* = 0.067).

The distribution of sociodemographic and training characteristics of PCPs by gender was shown in Table 1.

**Table 1:** Sociodemographic, training characteristics and experiences, attitudes of PCPs in terms of gender.

Variables		Male (*n* = 213) Mean ± SD and *n*(%)	Female (*n* = 241) Mean ± SD and *n*(%)	*p* value
Age (years)		37.80 ± 6.06	36.75 ± 8.32	0.131^a^
Duration of primary care practice (years)		7.83 ± 5.32	6.92 ± 5.78	0.084^a^
Specialization status	General practitioner	196 (92.0)	125 (51.9)	<0.001*^b^
	Family medicine specialist	17 (8.0)	116 (48.1)	
Place of working	Urban	95 (44.6)	104 (43.2)	0.756^b^
	Rural	118 (55.4)	137 (56.8)	
Psychiatry in-service training		101 (47.4)	133 (55.2)	0.098^b^
Using classification criteria	No	65 (30.5)	35 (14.5)	<0.001*^b^
	DSM	64 (30.0)	142 (58.9)	
	ICD	84 (39.4)	64 (26.6)	
Any mental diagnosis experience in primary care practice		201 (94.4)	232 (96.3)	0.336^b^
The most easily diagnosed mental disorder	Anxiety spectrum disorders	130 (61.0)	126 (52.3)	0.116^b^
	OCD spectrum	15 (7.0)	14 (5.8)	
	Depression spectrum disorders	56 (26.3)	89 (36.9)	
	Bipolar spectrum disorders	12 (5.6)	12 (5.0)	
The most difficult diagnosed mental disorder	OCD spectrum	16 (7.5)	15 (6.2)	0.366^b^
	Depression spectrum disorders	6 (2.8)	4 (1.7)	
	Bipolar spectrum disorders	124 (58.2)	159 (66.0)	
	Schizophrenia spectrum disorders	67 (31.5)	63 (26.1)	
The most difficult disorder in the follow-up and treatment process	Depression spectrum disorders	13 (6.1)	8 (3.3)	<0.001*^b^
	Bipolar spectrum disorders	62 (29.1)	59 (24.5)	
	Schizophrenia spectrum disorders	52 (24.4)	23 (9.5)	
	Alcohol/drug use disorders	86 (40.4)	151 (62.7)	
Approach to the psychotic patient	I refer directly to a psychiatrist	198 (93.0)	232 (96.3)	0.116^b^
	I start treatment and then refer a psychiatrist	15 (7.0)	9 (3.7)	

*p < 0.001. Independent sample *t*-test^a^ and Chi-square test^b^ were used in statistical analysis.

PCP, Primary care provider; SD, Standard deviation; DSM, Diagnostic and Statistical Manual of Mental Disorders; ICD, International Classification of Diseases; OCD, Obsessive-compulsive disorder.

### Clinical approaches and experiences of PCPs

In primary care practice, 267 PCPs (58.8%) considered a diagnosis of depression spectrum disorder in at least one patient; 378 PCPs (83.3%) considered a diagnosis of anxiety spectrum disorder in at least one patient; 271 PCPs (59.7%) considered a diagnosis of panic disorder in at least one patient; 156 PCPs (34.4%) considered an obsessive-compulsive spectrum disorder diagnosis in at least one patient; 12 PCPs (2.6%) considered a diagnosis of schizophrenia spectrum disorder in at least one patient; 53 PCPs (11.7%) considered a diagnosis of bipolar spectrum disorder in at least one patient; 156 PCPs (34.4%) considered a diagnosis of alcohol/drug use disorder in at least one patient; 108 PCPs (23.8%) considered a diagnosis of sexual function disorder in at least one patient; 109 PCPs (24.0%) considered a diagnosis of attention-deficit hyperactivity disorder in at least one patient; 153 PCPs (33.7%) considered a diagnosis of postpartum depression in at least one patient.

Three hundred seventeen PCPs (69.8%) had encountered a request for a rest request report at least once. One hundred forty-eight PCPs (32.6%) had examined a patient with suicidal thoughts/behavior/attempts at least once. In patients presenting with insomnia complaints, 238 PCPs (52.4%) initiated the treatment themselves, 187 PCPs (41.2%) referred them to a psychiatrist, 13 PCPs referred them to neurology (2.86%), and 16 PCPs referred them to pulmonologists (3.52%).

The distribution of comfort with mental disorder diagnoses of PCPs and their approach to psychotic disorder by gender was shown in Table 1.

### Psychotropic use characteristics of PCPs

In primary care practice, 51 PCPs (11.2%) prescribed mood stabilizers to at least one patient; 116 PCPs (25.6%) had prescribed benzodiazepine to at least one patient; 32 PCPs (7.0%) had prescribed psychostimulants to at least one patient; 57 PCPs (12.6%) prescribed modafinil to at least one patient.

Two hundred ninety-one PCPs (64.1%) were prescribing all psychotropic medications reported by a psychiatrist, 78 PCPs (17.18%) were prescribing psychotropic medications other than red prescription drugs, and 29 PCPs (6.38%) were prescribing psychotropic medications other than red and green prescription drugs. Fifty-six PCPs (12.34%) did not prescribe psychotropic medications in any case.

The most frequently preferred benzodiazepines by 45 (9.9%) of PCPs were alprazolam, 33 (7.3%) were medazepam, 12 (2.6%) were diazepam, seven (1.5%) were lorazepam, three (0.7%) were clonazepam. Eighty-nine PCPs (19.6%) had experience prescribing benzodiazepines in the anxiety spectrum disorder.

Psychotropic use characteristics and attitudes of PCPs by gender were shown in Table 2.

**Table 2:** Psychotropic use characteristics and attitudes of PCPs in terms of gender.

Variables		Male (*n* = 213) Mean ± SD and *n*(%)	Female (*n* = 241) Mean ± SD and *n*(%)	*p* value
Thoughts on minimum duration for which ADs should be used (month)		5.42 ± 3.79	4.82 ± 3.00	0.061^a^
Thoughts on AD effect onset time (week)		2.44 ± 1.01	2.40 ± 1.51	0.748^a^
AD use experience		172 (80.8)	197 (81.7)	0.787^b^
AP use experience		27 (12.7)	44 (18.3)	0.102^b^
Medication preference in insomnia	I do not start treatment myself for insomnia	12 (5.6)	40 (16.6)	<0.001*^b^
	Quetiapine	14 (6.6)	23 (9.5)	
	Antihistamines	109 (51.2)	92 (38.2)	
	Medazepam	15 (7.0)	9 (3.7)	
	Trazodone	17 (8.0)	14 (5.8)	
	Opipramol	32 (15.0)	29 (12.0)	
	Herbal supplement	14 (6.6)	34 (14.1)	
First AD preference in depression spectrum disorders	I do not start treatment myself	63 (29.6)	23 (9.5)	<0.001*^b^
	Sertraline	74 (34.7)	119 (49.4)	
	Escitalopram	66 (31.0)	92 (38.2)	
	Citalopram	10 (4.7)	7 (2.9)	
First AD preference in anxiety disorder spectrum	I do not start treatment myself	78 (36.6)	63 (26.1)	<0.001*^b^
	Sertraline	21 (9.9)	55 (22.8)	
	Escitalopram	52 (24.4)	73 (30.3)	
	Citalopram	16 (7.5)	6 (2.5)	
	Paroxetine	28 (13.1)	17 (7.1)	
	Fluoxetine	18 (8.5)	27 (11.2)	
First AD preference in OCD spectrum	I do not start treatment myself	130 (61.0)	145 (60.2)	0.124^b^
	Sertraline	16 (7.5)	24 (10.0)	
	Escitalopram	10 (4.7)	17 (7.1)	
	Citalopram	11 (5.2)	5 (2.1)	
	Paroxetine	14 (6.6)	23 (9.5)	
	Fluoxetine	4 (1.9)	8 (3.3)	
	Clomipramine	6 (2.8)	7 (2.9)	
	Duloxetine	22 (10.3)	12 (5.0)	
Did you know that ADs must be used regularly every day for full effectiveness?		185 (86.9)	216 (89.6)	0.359^b^
Do ADs cause forgetfulness?		112 (52.6)	156 (64.7)	0.009*^b^
Do ADs cause numb?		104 (48.8)	113 (49.9)	0.680^b^
Do ADs lead to suicide?		12 (5.6)	13 (5.4)	0.911^b^
Are ADs addictive?		50 (23.5)	31 (12.9)	0.003*^b^
Implementing reported/prescribed LAI AP in family health center		177 (83.1)	167 (69.3)	0.001*^b^

*p < 0.001; Independent sample *t*-test^a^ and Chi-square test^b^ were used in statistical analysis; PCP, Primary care provider; SD, Standard deviation; AD, Antidepressant; AP, Antipsychotic; OCD, Obsessive-compulsive disorder; LAI, Long-acting injectable.

### Comparison of training characteristics, experiences and attitudes of PCPs in terms of gender

Experience of depression spectrum disorder diagnosis (*p* = 0.077), anxiety spectrum disorder diagnosis experience (*p* = 0.178), experience of panic disorder diagnosis (*p* = 0.584), obsessive-compulsive spectrum disorder diagnosis experience (*p* = 0.814), experience of schizophrenia spectrum disorder diagnosis (*p* = 0.165), bipolar spectrum disorder diagnosis experience (*p* = 0.154), experience of alcohol/drug use disorder diagnosis (*p* = 0.105), sexual dysfunction diagnosis experience (*p* = 0.607), attention-deficit/hyperactivity disorder diagnosis experience (*p* = 0.529), antidepressant use experience (*p* = 0.787) and antipsychotic use experience (*p* = 0.102) were similar between genders. Postpartum depression diagnosis experience was higher in female PCPs (*p* < 0.001). Comparison of experiences and attitudes of PCPs according to gender was shown in Table 1.

### Comparison of training characteristics, experiences and attitudes of PCPs in terms of family medicine specialty

The rate of panic disorder diagnosis experience (*p* = 0.009) among family medicine specialists is higher than that of general practitioners. The diagnosis experience of depression spectrum disorder (*p* = 0.870), schizophrenia spectrum disorder (*p* = 0.106), bipolar spectrum disorder (*p* = 0.427), attention-deficit/hyperactivity disorder (*p* = 0.479), postpartum depression (*p* = 0.137), and anxiety spectrum disorder (*p* = 0.839) were similar between family medicine specialists and general practitioners.

The experience of using antidepressants (*p* = 0.615), mood stabilizers (*p* = 0.107), and the most frequently preferred benzodiazepine (*p* = 0.838) was similar between family medicine specialists and general practitioners. Experience in using antipsychotics (*p* < 0.001), benzodiazepines (*p* < 0.001), psychostimulants (*p* = 0.010), and modafinil (*p* = 0.001) was higher in general practitioners than in family medicine specialists.

The comparison of PCPs according to their specialization status in terms of various variables was shown in Tables 3, 4.

**Table 3:** Sociodemographic, training characteristics and experiences, attitudes of PCPs in terms of specialization status.

Variables		General practitioner (*n* = 321) Mean ± SD and *n*(%)	Family medicine specialist (*n* = 133) Mean ± SD and *n*(%)	*p* value
Age (years)		37.94 ± 7.18	35.56 ± 7.55	0.002*^a^
Duration of primary care practice (years)		8.06 ± 6.05	5.61 ± 3.74	<0.001**^a^
Psychiatry in-service training		129 (40.2)	105 (78.9)	<0.001**^b^
Number of daily patient examinations	0–20 examination	99 (30.8)	49 (36.8)	0.175^b^
	21–50 examination	118 (36.8)	37 (27.8)	
	>51 examination	104 (32.4)	47 (35.3)	
Use of classification criteria	No	100 (31.2)	0 (0.0)	<0.001**^b^
	DSM	81 (25.2)	125 (94.0)	
	ICD	140 (43.6)	8 (6.0)	
Any mental diagnosis experience in primary care practice		300 (93.5)	133 (100.0)	0.003*^b^
The most easily diagnosed mental disorder	Anxiety spectrum disorders	209 (65.1)	47 (35.3)	<0.001**^b^
	OCD spectrum	26 (8.1)	3 (2.3)	
	Depression spectrum disorders	69 (21.5)	76 (57.1)	
	Bipolar spectrum disorders	17 (5.3)	7 (5.3)	
The most difficult diagnosed mental disorder	OCD spectrum	24 (7.5)	7 (5.3)	0.395^b^
	Depression spectrum disorders	5 (1.6)	5 (3.8)	
	Bipolar spectrum disorders	198 (61.7)	85 (63.9)	
	Schizophrenia spectrum disorders	94 (29.3)	36 (27.1)	
The most difficult disorder in the follow-up and treatment process	Depression spectrum disorders	17 (5.3)	4 (3.0)	<0.001**^b^
	Bipolar spectrum disorders	88 (27.4)	33 (24.8)	
	Schizophrenia spectrum disorders	69 (21.5)	6 (4.5)	
	Alcohol/drug use disorders	147 (45.8)	90 (67.7)	
Approach to the psychotic patient	I refer directly to a psychiatrist	297 (92.5)	133 (100.0)	0.001*^b^
	I start treatment and then refer a psychiatrist	24 (7.5)	0 (0.0)	

*p < 0.05, **p < 0.001. Independent sample *t*-test^a^ and Chi-square test^b^ were used in statistical analysis.

PCP, Primary care provider; SD, Standard deviation; DSM, Diagnostic and Statistical Manual of Mental Disorders; ICD, International Classification of Diseases; OCD, Obsessive-compulsive disorder.

**Table 4:** Psychotropic use characteristics and attitudes of PCPs in terms of specialization status.

Variables		General practitioner (*n* = 321) Mean ± SD and *n*(%)	Family medicine specialist (*n* = 133) Mean ± SD and *n*(%)	*p* value
Thoughts on minimum duration for which ADs should be used (month)		5.28 ± 3.55	4.66 ± 2.98	0.077^a^
Thoughts on AD effect onset time (week)		2.27 ± 1.17	2.79 ± 1.50	<0.001**^a^
AD use experience		259 (80.7)	110 (82.7)	0.615^b^
AP use experience		63 (19.6)	8 (6.0)	<0.001**^b^
Mood stabilizer use experience		41 (12.8)	10 (7.5)	0.107^b^
BZD use experience		98 (30.5)	18 (13.5)	<0.001**^b^
Psychostimulant use experience		29 (9.0)	3 (2.3)	0.010*^b^
Modafinil use experience		51 (15.9)	6 (4.5)	0.001*^b^
Medication preference in insomnia	I do not start treatment myself for insomnia	52 (16.2)	0 (0.0)	<0.001**^b^
	Quetiapine	14 (4.4)	23 (17.3)	
	Antihistamines	113 (35.2)	88 (66.2)	
	Medazepam	16 (5.0)	8 (6.0)	
	Trazodone	31 (9.7)	0 (0.0)	
	Opipramol	56 (17.4)	5 (3.8)	
	Herbal supplement	39 (12.1)	9 (6.8)	
First AD preference in depression spectrum disorders	I do not start treatment myself	86 (26.8)	0 (0.0)	<0.001**^b^
	Sertraline	105 (32.7)	88 (66.2)	
	Escitalopram	115 (35.8)	43 (32.3)	
	Citalopram	15 (4.7)	2 (1.5)	
First AD preference in anxiety spectrum disorders	I do not start treatment myself	108 (33.6)	33 (24.8)	<0.001**^b^
	Sertraline	46 (14.3)	30 (22.6)	
	Escitalopram	74 (23.1)	51 (38.3)	
	Citalopram	19 (5.9)	3 (2.3)	
	Paroxetine	42 (13.1)	3 (2.3)	
	Fluoxetine	32 (10.0)	13 (9.8)	
First AD preference in OCD spectrum	I do not start treatment myself	184 (57.3)	91 (68.4)	<0.001**^b^
	Sertraline	16 (5.0)	24 (18.0)	
	Escitalopram	15 (4.7)	12 (9.0)	
	Citalopram	13 (4.0)	3 (2.3)	
	Paroxetine	37 (11.5)	0 (0.0)	
	Fluoxetine	11 (3.4)	1 (0.8)	
	Clomipramine	13 (4.0)	0 (0.0)	
	Duloxetine	32 (10.0)	2 (1.5)	
The most frequently experienced psychotropic side effect	I did not encounter	48 (15.0)	0 (0.0)	<0.001**^b^
	Sedation	171 (53.3)	97 (72.9)	
	Dystonia	24 (7.5)	0 (0.0)	
	Constipation	19 (5.9)	0 (0.0)	
	Mouth dry	12 (3.7)	13 (9.8)	
	Palpitation	47 (14.6)	0 (0.0)	
	Headache/dizziness	0 (0.0)	23 (17.3)	
Did you know that ADs must be used regularly every day for full effectiveness?		268 (83.5)	133 (100.0)	<0.001**^b^
Do ADs cause forgetfulness?		195 (60.7)	73 (54.9)	0.248^b^
Do ADs cause numb?		171 (53.3)	46 (34.6)	<0.001**^b^
Do ADs lead to suicide?		25 (7.8)	0 (0.0)	0.001*^b^
Are ADs addictive?		58 (18.1)	23 (17.3)	0.844^b^
Implementing reported/prescribed LAI AP in family health center		291 (90.7)	53 (39.8)	<0.001**^b^

*p < 0.05, **p < 0.001. Independent sample *t*-test^a^ and Chi-square test^b^ were used in statistical analysis.

PCP, Primary care provider; SD, Standard deviation; AD, Antidepressant; AP, Antipsychotic; BZD, Benzodiazepine; OCD, Obsessive-compulsive disorder; LAI, Long-acting injectable.

### Comparison of training characteristics, experiences and attitudes of PCPs in terms of in-service training in psychiatry

The rate of receiving in-service training in psychiatry (*p* < 0.001) was significantly higher in family medicine specialists than in general practitioners. The rate of using DSM as a basis when evaluating mental disorders (*p* < 0.001) was higher in PCPs who received in-service training in psychiatry. Anxiety spectrum disorder (*p* < 0.001), panic disorder (*p* = 0.018), obsessive-compulsive spectrum disorder (*p* < 0.001), bipolar spectrum disorder (*p* < 0.001), sexual function disorder (*p* < 0.001), attention-deficit/hyperactivity disorder (*p* < 0.001) and postpartum depression (*p* < 0.001) diagnosis experience was significantly higher in PCPs who received in-service training in psychiatry.

Experience in using antidepressants (*p* < 0.001), antipsychotics (*p* < 0.001), mood stabilizers (*p* < 0.001) and benzodiazepines (*p* = 0.001) was significantly higher in PCPs who received in-service training in psychiatry.

PCPs, who had not received in-service training in psychiatry, referred all patients presenting with psychotic symptoms to psychiatry. The experience of encountering suicide-related situations was significantly higher in PCPs who received in-service training in psychiatry (*p* < 0.001). The rate of starting treatment in insomnia (*p* < 0.001), depression spectrum disorder (*p* < 0.001), anxiety spectrum disorder (*p* < 0.001), and obsessive-compulsive spectrum disorder (*p* < 0.001) was higher in PCPs who received in-service training in psychiatry (*p* < 0.001). The false belief that antidepressants cause suicide and addiction was higher in PCPs who did not receive in-service training in psychiatry.

### Application of binary logistic regression analysis to various variables

Binary logistic regression analysis was used to predict specialization status and was applied separately for each significant independent variable. According to the binary logistic regression analysis, the *p* values of psychiatry in-service training, the most easily diagnosed mental disorder, the most difficult mental disorder in the follow-up and treatment process, medication preference in insomnia, and regular daily use of antidepressants were determined to be less than 0.05. According to the binary logistic regression analysis, the sensitivity of our model was 93.1, and the specificity was 98.5% (Beginning block −2 Log likelihood 549.399, overall *p* value < 0.001; Block one −2 Log likelihood 99.417^a^, Cox & Snell *R*^2^ = 0.629, Nagelkerke *R*^2^ = 0.896; Hosmer and Lemeshow Test *p* value > 0.999).

Binary logistic regression analysis was used to predict psychiatry in-service training experience and was applied separately for each significant independent variable. While creating the model, the variables with the highest significance level under the same topic (e.g., only one of the variables such as antidepressant use experience, antipsychotic use experience, benzodiazepine use experience) were included. Also, it was aimed to reach the highest significance level with fewer variables. According to the binary logistic regression analysis, the *p* values of using classification criteria, experience of anxiety spectrum disorder diagnosis, antidepressant use experience, approach to psychotic disorder, suicide detection experience, first antidepressant choice in anxiety, and addictive status of antidepressants were determined to be less than 0.05. Anxiety disorder diagnosis experience, antidepressant use experience, and approach to psychotic disorder variables were excluded because they did not contribute sufficiently to the model. According to the binary logistic regression analysis, the sensitivity of our model (using classification criteria, suicide detection experience, first antidepressant choice in anxiety, addictive status of antidepressants) was 88.6, and the specificity was 98.3% (Beginning block −2 Log likelihood 628.946, overall *p* value < 0.001; Block one −2 Log likelihood 141.054^a^, Cox & Snell *R*^2^ = 0.659, Nagelkerke *R*^2^ = 0.878; Hosmer and Lemeshow Test *p* value 0.938).

## Discussion

This study examined the experiences, practices, and attitudes of PCPs in Turkey regarding mental disorder management. PCPs differed significantly in their approach to mental disorders and in their psychotropic selection. Gender was found to be associated with various variables: the majority of family medicine specialists are females. This disparity influenced various comparison findings between genders. The findings need to be interpreted in light of this information.

When the findings of our study are examined, it can be seen that almost all the PCPs had experience in considering a diagnosis of a mental disorder in at least one patient. The majority of common mental disorders visit PCPs. Anxiety and depression spectrum disorders were the most frequently encountered and most easily diagnosed mental disorders in primary care practice. This finding appears to be compatible with the literature (23). Schizophrenia and bipolar spectrum disorders have also been identified as mental disorders for which PCPs experience difficulties in the diagnosis and treatment process. The general approach is that PCPs recognize psychotic and affective symptoms and refer these individuals to psychiatry. It is thought that bipolar and schizophrenia spectrum disorders, which have been diagnosed and are in remission, can be followed-up by PCPs (24, 25). According to this study, suicide-related situations were experienced by one third of PCPs. However, the real number is thought to be higher than this. Unfortunately, inadequate training also manifests itself in the process of handling suicide. Inquiring about suicide-related situations, which is an essential part of mental history-taking, is often skipped or not handled appropriately. This situation may cause many cases to be overlooked (26). To solve these problems, mental status examination should be discussed in detail in in-service training in psychiatry, and the importance of inquiring vegetative symptoms such as sleep and appetite and suicide-related situations should be emphasized (20).

Specialization status appears to be reflected in the results. Family medicine specialists had more psychiatry in-service training experience. This is likely to be related to local Ministry of Health policies. In-service training related to psychiatry may be aimed primarily at training family medicine specialists. In the survey, the opinions of general practitioners and family medicine specialists differed regarding mental disorders that are most easily diagnosed and most difficult to follow-up and treat. Family medicine specialists reported that the mental disorder they had the most difficulty in monitoring and treating was alcohol/drug use disorders. It is thought that the possible reason for this may be related to the content of psychiatry in-service training. In recent years, serious studies have been carried out in Turkey regarding the follow-up and treatment of alcohol/drug use disorders (27). These studies may be carried out by family medicine specialists through psychiatry in-service training. Both general practitioners and family medicine specialists reported that the type of mental disorder they found most difficult to diagnose was bipolar spectrum disorders. Bipolar spectrum disorders is often confused with conditions such as major depressive disorder, borderline personality disorder, schizophrenia, attention-deficit/hyperactivity disorder, or adolescence, and differential diagnosis requires serious effort and knowledge (28–30). The early identification of bipolar spectrum disorders is problematic. Patients with bipolar spectrum disorder symptoms may be misdiagnosed and improperly treated, resulting in significant treatment failure. In the primary care setting, patients who have bipolar spectrum disorder may represent a significant proportion of the difficult-to-treat depressed and anxious patients (28). PCPs can be trained on the differential diagnosis of bipolar spectrum disorder or indications for referral to psychiatry through psychiatry in-service training. Otherwise, a bipolar spectrum disorder diagnosis may be missed for several years or longer (28, 31).

Several epidemiological studies have clearly shown that primary and secondary sleep disturbances are very common in the general population. Common complaints include difficulty falling and staying asleep, frequent awakenings, and excessive daytime sleepiness (32). Insomnia is among the most common sleep disorder symptoms (33). Although insomnia is treatable, it often remain underdiagnosed. If insomnia is left untreated, its negative impact on daytime functioning may be challenging (32, 33). It is important for physicians to be aware of it, as it causes considerable physical and psychological stress. In this study, the medication preferences of PCPs regarding insomnia were elicited and our findings show that PCPs hesitate to initiate treatment for insomnia. Half of PCPs refer patients with insomnia to various medical specialties including psychiatry, neurology, and pulmonology. PCPs who initiate treatment often prefer antihistamines. PCPs tend to use medications with less sleep-inducing effects in the treatment of insomnia. This can be interpreted as PCPs not wanting to encounter possible psychotropic side effects. However, it is possible that uncontrolled use of antihistamines for a prolonged period may cause various problems (34). This is where in-service training in psychiatry plays an important role. In these trainings, PCPs, whose knowledge level is increased about sleep disorders, the distinction between primary and secondary sleep disorders, ensuring sleep hygiene, medications that can be used and possible side effects, can complete the treatment of these patients in primary care and prevent the patients from occupying secondary and tertiary healthcare institutions.

It seems that knowledge of PCPs about psychotropic medications is not sufficient. As with patients and their relatives, the number of PCPs who thought that antidepressants were addictive and caused suicide, numbness and forgetfulness was not small (35, 36). One eighth of PCPs did not prescribe psychotropic medications under any circumstances. Experience with psychotropic use and knowledge about the general characteristics of antidepressants vary significantly among PCPs. Experience in prescribing antipsychotics, psychostimulants, and modafinil was higher among general practitioners. Although the rate of in-service training in psychiatry was lower, general practitioners have a greater variety of psychotropic drug use. Under normal circumstances, those who receive in-service training would be expected to report a higher variety of psychotropic use. This situation was interpreted as the need to reorganize the content of in-service training in psychiatry. Another important finding of this study is that those who have not received in-service training in psychiatry do not take into account classification systems, when evaluating mental disorders and are far from employing a systematic approach. It is recommended to include clinical pharmacists in the in-service training in psychiatry trainer group when creating psychiatry training curricula (37, 38). PCPs should be informed about drug interactions with the contribution of clinical pharmacists, and approaches for special groups such as elderly patients should be highlighted. Aging is associated with progressive decrease in the function of multiple organs, presence of comorbidities, polypharmacy, and social and functional problems that may lead to inappropriate use of medications (39). Psychotropics are widely used in the elderly population and often their use is inappropriate. Inappropriate use of psychotropic medications has been associated with a high incidence of side effects especially in the older population such as cognitive and psychomotor impairment, falls and fractures (40). In order to reduce inappropriate use of psychotropic medications in elderly, it is important to stay away from polypharmacy, manage with the optimum effective dose, closely monitor the functions of organs such as the liver and kidney, and keep the time between visits short. Psychiatry training of PCPs addressing the use of psychotropic medications in elderly patients should have these features (39).

Patients who were thought to have psychotic symptoms were referred to a psychiatrist by PCPs who received in-service training in psychiatry. Experience of encountering suicide-related situations was also higher among PCPs who received in-service training in psychiatry. A PCP who has no or insufficient knowledge about a subject cannot be expected to question on it. In this sense, it is meaningful that PCPs who receive in-service training in psychiatry have higher experience with patients with suicide ideation and psychotic symptoms. The rate of starting treatment for various mental conditions was also higher among PCPs who received in-service training in psychiatry. Misinformation about antidepressants was also more common among PCPs who did not receive in-service training in psychiatry.

It is thought that many mental disorders, especially anxiety and depression spectrum disorders, can be diagnosed and treated effectively by PCPs. The World Health Organization and American Psychiatric Association developed specific diagnostic guidelines for the mental disorders in primary care (41). A significant portion of symptoms of mental disorders can be treated with a medical education that understands the importance of the subject and in-service training in psychiatry repeated at regular intervals. As in many parts of the world, there are studies examining psychiatry education in medicine and its sub-branches in Turkey (42). A study was conducted by Çıngı-Başterzi et al. (42) in 2007 in the psychiatry departments of 29 of 59 medical schools in Turkey. In this study (42), it was shown that the Core Education Program created by Turkish Medicine and Health Education Council was implemented in only 37.5% of medical schools, that preclinical and clinical training contents varied between medical schools, and that three of the medical schools did not offer internships in psychiatry. The majority of chairs of psychiatry departments emphasized the importance of mood disorders (49.9%) and anxiety disorders (40%), suggesting that these disorders should be treated by general practitioners (42). Çıngı-Başterzi et al. (42) suggested that a standardized curriculum should be developed in line with international guidelines for psychiatric education. There is no more up-to-date source examining the psychiatry training of medical students and PCPs in Turkey. Newer studies are needed to determine the possible situation and gaps. It is thought that the present study will contribute to filling this gap in Turkey.

Although this study examined the approach of PCPs in Turkey to mental disorders, there are problems arising from lack or inadequacy of training in many countries (43, 44). Based on the findings of this study, the approaches and experiences of PCPs in various countries regarding mental disorders can be compared. Health care professionals need to work in collaboration in order to maintain standardized and internationally valid training based on these and similar studies. While the organization of a country’s healthcare system will differ according to the geography, healthcare priorities, availability of resources, and funding, many authorities have recognized the importance of collaboration between primary care services and specialized mental health to enable primary care to deliver effective mental health care. Indeed, the World Health Organization has recommended that integrating mental health services within primary care may be the optimal way of responding to the increasing demand for mental health care (45).

## Strengths, limitations, and future directions

The most conspicuous aspect of this study is that it examines the approaches and experiences of family medicine specialists and general practitioners regarding mental disorders, taking into account the psychiatry in-service training factor. In this study, approaches to diagnosis and treatment of mental disorders were examined together and in detail. The cross-sectional nature of the study can be considered a limitation. In addition, antidepressant subclasses were not questioned under separate headings, and all antidepressant medications were included under the general term ‘antidepressant’. Furthermore, PCP experiences with mental disorders were self-reported, and it was not possible to determine whether the information reported by PCPs was correct. The position of sociodemographic characteristics, clinical experiences, perspectives on such survey studies, and willingness to participate in surveys of the participants who filled out the surveys distributed to WhatsApp and Yahoo groups are not known within the PCP universe in Turkey, that similar limitations may also be encountered in face-to-face survey studies. Finally, their experience in diagnosing mental disorders was not verified by another healthcare professional. In this sense, prospective studies are needed.

## Conclusion

This study reports that approaches, experiences and knowledge levels of PCPs regarding the diagnosis and treatment of mental disorders show significant differences. It seems that one of the most important reasons for these differences is education. The handling of mental cases in medical education and the existence and content of in-service training in psychiatry after medical school graduation affect the mental knowledge and approaches of PCPs. First, the importance given to the diagnosis and treatment of mental disorders in medical education needs to be emphasized, and theoretical knowledge needs to be supported practically. Afterwards, all PCPs are required to update their knowledge at regular intervals through in-service training in psychiatry.

## Data availability statement

The raw data supporting the conclusions of this article will be made available by the authors, without undue reservation.

## Ethics statement

The studies involving humans were approved by Fırat University Non-invasive Research Ethics Committee: Date: 14/09/2023; Number: 2023/12-12. The studies were conducted in accordance with the local legislation and institutional requirements. The participants provided their written informed consent to participate in this study.

## Author contributions

DÖ: Conceptualization, Data curation, Formal analysis, Funding acquisition, Investigation, Methodology, Project administration, Resources, Software, Supervision, Validation, Visualization, Writing – original draft, Writing – review & editing.
